# Microbial diversity in a Venezuelan orthoquartzite cave is dominated by the *Chloroflexi* (Class *Ktedonobacterales*) and *Thaumarchaeota* Group I.1c

**DOI:** 10.3389/fmicb.2014.00615

**Published:** 2014-11-26

**Authors:** Hazel A. Barton, Juan G. Giarrizzo, Paula Suarez, Charles E. Robertson, Mark J. Broering, Eric D. Banks, Parag A. Vaishampayan, Kasthisuri Venkateswaran

**Affiliations:** ^1^Department of Biology and Department of Geosciences, University of AkronAkron, OH, USA; ^2^Department of Biological Sciences, Northern Kentucky University, Highland HeightsKY, USA; ^3^Departamento de Biología de Organismos, Universidad Simón BolívarCaracas, Venezuela; ^4^Department of Molecular, Cellular and Developmental Biology, University of Colorado, BoulderCO, USA; ^5^Biotechnology and Planetary Protection Group, Jet Propulsion Laboratory, California Institute of TechnologyPasadena, CA, USA

**Keywords:** orthoquartzite, cave, *Ktedonobacterales*, *Thaumarchaeota*, geomicrobiology

## Abstract

The majority of caves are formed within limestone rock and hence our understanding of cave microbiology comes from carbonate-buffered systems. In this paper, we describe the microbial diversity of Roraima Sur Cave (RSC), an orthoquartzite (SiO_4_) cave within Roraima Tepui, Venezuela. The cave contains a high level of microbial activity when compared with other cave systems, as determined by an ATP-based luminescence assay and cell counting. Molecular phylogenetic analysis of microbial diversity within the cave demonstrates the dominance of *Actinomycetales* and *Alphaproteobacteria* in endolithic bacterial communities close to the entrance, while communities from deeper in the cave are dominated (82–84%) by a unique clade of *Ktedonobacterales* within the *Chloroflexi*. While members of this phylum are commonly found in caves, this is the first identification of members of the Class *Ktedonobacterales*. An assessment of archaeal species demonstrates the dominance of phylotypes from the *Thaumarchaeota* Group I.1c (100%), which have previously been associated with acidic environments. While the *Thaumarchaeota* have been seen in numerous cave systems, the dominance of Group I.1c in RSC is unique and a departure from the traditional archaeal community structure. Geochemical analysis of the cave environment suggests that water entering the cave, rather than the nutrient-limited orthoquartzite rock, provides the carbon and energy necessary for microbial community growth and subsistence, while the poor buffering capacity of quartzite or the low pH of the environment may be selecting for this unusual community structure. Together these data suggest that pH, imparted by the geochemistry of the host rock, can play as important a role in niche-differentiation in caves as in other environmental systems.

## INTRODUCTION

The majority of caves form in soluble rock such as limestone, a sedimentary rock mainly comprised of calcium carbonate (Klimchouk et al., 2000). Classic cave formation, or speleogenesis, normally occurs through the chemical dissolution of this rock by water, which becomes acidified via carbonic acid when passing through CO_2_-rich soils. Occasionally microbially derived acids, such as sulfuric acid, can also lead to the dissolution of caves (Palmer and Palmer, 2000; Klimchouk, 2007; Barton, 2013). Once formed, caves provide a unique portal into the deep subsurface (up to ∼2,000 m) in which to study geomicrobial interactions and processes under relatively stable conditions. As most caves are formed in limestone the majority of microbiology carried out in caves has been described in such systems (Sarbu et al., 1996; Angert et al., 1998; Groth et al., 2001; Northup et al., 2003; Chelius and Moore, 2004; Spear et al., 2007; Macalady et al., 2008; Banks et al., 2010; Bhullar et al., 2012; Cuezva et al., 2012; Barton, 2014). Such studies demonstrate microbial species often adapted to oligotrophy, with a dominance of *Alpha*- and *Betaproteobacteria*, presumably involved in nitrogen fixation, along with significant populations of *Firmicutes* and *Actinobacteria*, suggesting an important role for heterotrophic interactions and carbon turnover (Barton, 2014). Nonetheless, when deep-sequencing technologies are used to examine these environments, the results suggest that there is much to learn about the depth and breadth of microbial physiology in caves (Tetu et al., 2013; Ortiz et al., 2014).

The insoluble, glass-like nature of orthoquartzite (a quartz-cemented sandstone) makes it resistant to the weathering processes that routinely form caves; sandstone caves are traditionally shallow, near-surface features formed via aeolian or tectonic processes (Turkington and Paradise, 2005); however, tropical sandstones demonstrate karst-like solution features. The recent exploration within the Tepui Mountains of Venezuela has identified a large number of caves, including some of the longest and deepest sandstone caves in the world (Wray, 1997; Auler, 2004; Aubrecht et al., 2008, 2011; Smida et al., 2008; Piccini and Mecchia, 2009). The exact mechanism of this cave formation remains unclear, although it appears that the water-mediated dissolution of the quartz cements plays an important role, as evidenced by the formation of sandy sediments on surfaces within the cave (Piccini and Mecchia, 2009; Aubrecht et al., 2011). As orthoquartzite rock remains relatively impermeable to the movement of water, this dissolution appears to occur via surface water penetrating unconsilidated layers within the rock massif (Piccini and Mecchia, 2009; Aubrecht et al., 2011). As such, these caves would be considered ‘karst’ systems in the traditional sense, although their morphology appears to be unique to the Tepui mountains of Venezuela and Brazil (Aubrecht et al., 2011).

**FIGURE 1:**
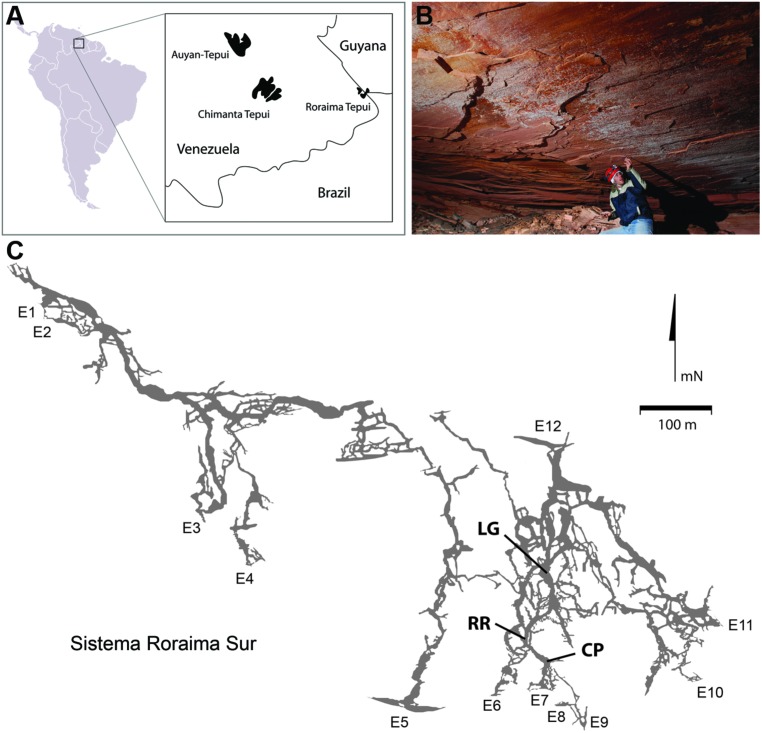
**(A)** Geographic location of Roraima Tepui; **(B)** Microbial colonies are present across the ceilings in locations within the cave; the microbial colonies are obvious as white markings against the pink/red color of the orthoquartzite; **(C)** Map of Roraima Sur Cave, showing the extent of the 16 km cave system, including the location of the three sampling sites used. Map used with permission from the University of Oxford Speleological Society and the Sociedad Venezolana de Espeleología.

Roraima Tepui, a 2,700 m high massif consisting of quartz (SiO_2_) cemented horizontal and gently dipping fluvial sandstones (quartz arenites), is located at the intersection of Venezuela, Guyana and Brazil (**Figure 1**; Briceno et al., 1990; Santos et al., 2003). The surface of the Tepui demonstrates extensive microbial colonization that has dramatically changed the landscape, covering exposed surfaces with a thick (mm–cm) characteristically black endolithic community comprised of *Cyanobacteria* and fungi (Gorbushina et al., 2001). Located within this massif is Roraima Sur Cave (RSC; aka Cueva Ojos de Cristal; **Figure 1**), one of the longest quartzite caves yet described at over 16 km in length (Galan et al., 2004; Smida et al., 2008).

Due to the limited weathering and the poor nutrient availability of orthoquartzite, the Tepui mountains are often bare or covered only with a thin soil (Maguire, 1970; Allen and Hajek, 1989; Michelangeli, 2000). In the absence of significant soils, surface ecosystems are nitrogen limited, which has led to an abundance of carnivorous plants in the local flora (Maguire, 1970; Steyermark, 1979). Due to the poor buffering capacity (when compared to carbonates) and the limited nutrient availability of orthoquartzite, we anticipated that any microbial activity within RSC would be minimal. Nonetheless, during a reconnaissance trip significant microbial activity was observed on exposed surfaces within the cave (**Figure 1B**) and appeared to be linked to a stream flowing through the cave. Examining these microbial communities using molecular techniques demonstrated that the cave contains an unusual microbial ecosystem dominated by both members of the *Chloroflexi* (Class *Ktedonobacterales*) and *Thaumarchaeota*, and is unlike any previous community described in carbonate caves (Northup et al., 2003; Chelius and Moore, 2004; Spear et al., 2007; Tetu et al., 2013; Barton, 2014; Ortiz et al., 2014). Our results suggest that nitrogen and the poor buffering by quartz may distinguish the microbial communities of sandstone caves from comparative carbonate systems.

## MATERIALS AND METHODS

### SAMPLE SITES AND ATP ANALYSES

Roraima Sur Cave is located at the end of a surface sinkhole on Roraima Tepui that takes a stream draining through surface vegetation before entering the cave. This water flows into the cave at ∼0.5–2.0 m^3^ s^-1^ depending on rainfall. Three sampling sites were used within the cave, which appeared to representative of an ‘average’ surface (did not contain any obvious microbial growth) and were 55, 90, and 300 m from the cave entrance (**Figure 1**). These three sites were: Cricket Pool (CP), a ceiling site ∼2.5 m above a still pool in which foraging crickets have been observed (P. Sprouse, personal communication, 2005); Red River (RR), a ceiling site in a 1.5 m high paleo-passage that is characterized by the high abundance of iron minerals; and Lago Grande (LG), located on a wall ∼2 m away from the largest lake within the cave. Due to the ongoing speleogenesis of the cave, all sampled surfaces were coated with unconsolidated, sandy sediments (Aubrecht et al., 2011). Approximately 10 *g* of these sediments collected for analysis at each sample site within the cave in January 2007. Ceiling and wall sediments were collected using a sterile scoop. Control samples were collected from outside of the cave entrance from areas without obvious *Cyanobacterial* growth; however, this material had not undergone the same erosional processes as the cave samples and remained in its cemented, rock-like state. Samples for DNA extraction were stored in 70% ethanol and kept at 4°C until arrival in the lab, whereupon samples were frozen at -80°C. Each site was swabbed for ATP by swabbing an ∼2 cm^2^ area using a portable luminescent Checklite-HS ATP assay (Kikkoman International, Noda, Japan; Venkateswaran et al., 2003).

### MICROSCOPY AND CELL COUNTS

Unless otherwise stated, all chemicals were obtained from Sigma Chemical (St. Louis, MO, USA). Samples for cell counting were fixed in 4% paraformaldehyde/phosphate buffered saline (PBS) on site for 4 h, followed by washing with PBS and storage in 50% methanol/PBS. Samples were kept at 4°C until arrival in the lab, whereupon samples were frozen at -20°C. For cell enumeration, 1 cm^3^ of sediment was washed in 1 × PBS and resuspended in 10 mL SYBR Green I/PBS for 15 min. To count cells, 10 μl of sample was then place on a microscope slide and examined under fluorescence on a Nikon Eclipse E600 microscope with a B-2E/C band-pass emission FITC filter and Remote Focus Z-stage controller (Nikon, Melville, NY, USA). An ocular grid of 100 μm^2^ at 1000 × magnification with a vertical range of 100 μm allowed the number of cells within a known volume of sediment (0.001 mm^3^) to be counted visually. The number of cells per cm^3^ of wall material for an average of nine observations was calculated as: average number of visualized cells × [1/volume measured (0.001 mm^3^)] × (1/dilution factor) × 1000]. For scanning electron microscopy (SEM) analysis, paraformaldehyde-fixed samples were washed in 70% ethanol/PBS, and dehydrated in an ethanol/PBS series to 100%. Samples were dried in a critical point dryer using liquid CO_2_ before examination under vacuum using a FEI Quanta 200 ESEM (Hillsboro, OR, USA).

### MOLECULAR TECHNIQUES

Genomic DNA was obtained from 1.5 *g* of cave sediment by first blocking the quartz with 2 μg of UV-irradiated polydI-dC (Barton et al., 2006), followed by the PowerSoil DNA Kit (MO BIO, Carlsbad, CA, USA). Even with crushing, we were unable to obtain amplifiable DNA from the rock-like surface control samples. To amplify the 16S ribosomal RNA gene sequence, a 40 μl PCR reaction containing 10 μl 2X *Taq* Master Mix (New England Biolabs, Ipswich, MA, USA; 10 mM Tris-HCl, 50 mM KCl, 1.5 mM MgCl_2_, 0.2 mM dNTPs, 5% Glycerol 0.08% NP-40 0.05% Tween-20, 0.5 units of *Taq* DNA Polymerase) ∼100 mM of each primer, and 50 ng of template gDNA was set up using the bacterial primers 8F (5′ – AGA GTT TGA TCM TGG CTC AG – 3′) and 1391R (5′– GAC GGG CGG TGW GTR CA – 3′; Spear et al., 2005). PCR amplification was carried out with a hot-start at 94°C for 8 min, followed by 30 s at 94°C, 45 s at 58°C and 1 min at 72°C for 30 cycles. This was followed by a elongation cycle at 72 °C for 8 min. For Archaeal sequences the Archaeal primers 4Fa (5′–TCC GGT TGA TCC TGC CRG- 3′) and 1100Ra (5′– TGG GTC TCG CTC GTT G-3′; Hales et al., 1996; Spear et al., 2005) were used with a 62°C annealing temperature. PCR products were purified with a ZR DNA Clean & Concentrator-25 Kit (Zymo Research, Orange, CA, USA) and cloned into a pTOPO-TA vector and transformed into competent *Escherichia coli* according to manufacturer’s protocol (Invitrogen, Carlsbad, CA, USA). Clones were picked and screened for unique phylotypes as previously described (Barton et al., 2004). Sanger sequencing of the clones was carried out by Agencourt Bioscience, Beverly, MA, USA and assembled together using DNA Baser software, obtaining minimally a 3X coverage for each examined sequence (and in practice at least a 6X coverage for the majority of clones). Assembled sequences were aligned and chimeras removed using the Greengenes NAST algorithm^1^. All sequences were submitted to the NCBI Genbank database under accession numbers GU205277–GU205318 (bacterial sequences) and KM214004–KM214181 (archaeal sequences).

### CONSTRUCTION OF PHYLOGENIES

Phylogenetic trees were built using backbone sequences from both the Ribosomal Database Project (RDP; Cole et al., 2005) and SILVA (Quast et al., 2013) databases and amended with additional sequences from the Genbank database^2^ as described (see Figure Legends). All sequences were aligned using the ARB software package version 5.1 (Ludwig et al., 2004) with fine scale alignment generated manually. Gaps were collapsed and the sequences were trimmed in ClustalW (Larkin et al., 2007). The phylogenetic relationship of 1276 (bacteria) and 774 (archaea) aligned bases of sequence data were determined using the maximum likelihood algorithm for 1000 bootstrap replicates using the RAxML Blackbox software (Stamatakis et al., 2008) in the CIPRES gateway (Miller et al., 2010). The model used and relevant outgroups are shown in each figure. FigTree version 1.4.1^3^ was used to prepare the phylogenetic trees, which were prepared for publication in with Adobe Illustrator CS5.

### PHYSICAL PARAMETERS AND GEOCHEMISTRY

As a rough estimate of available ammonia, nitrate and nitrite and pH at each sample site, 10 cm^3^ of rock was added to 10 mL deionized water (0 mg/L ammonia, 0 mg/L nitrite, 0 mg/L nitrite, and pH 6.9) and shaken briefly. The particulates were allowed to settle and the supernatant was tested for nitrogenous compounds with a field-available assay (Mardel Laboratories, Inc) with a detection limit of 0.50 mg/L for nitrate, 0.25 mg/L for nitrite, and 0.25 mg/L for ammonia. Total dissolved silica was determined using a Hach Portable Colorimeter II using the silicomolybdate method (Knudson et al., 1940; Hach, Loveland, CO, USA), while pH was measured using an Accumet AP61 portable pH meter (Fisher Scientific, Pittsburg, PA, USA). Relative humidity (RH) and temperature were measured in the cave using a RH300 Psychrometer (Extech instruments, Waltham, MA, USA). For elemental analysis, samples were crushed and examined via X-ray fluorescence in a Bruker GmbH S4 Pioneer-4kW wave-length dispersive X-ray spectrometer (Billerica, MA, USA). The Mossbauer spectrum was measured using a conventional constant acceleration-driving unit from Halder, GmbH (Bad Waldsee, Germany), connected to a 386 personal computer by a Canberra Nuclear PHA/MCS interface card (Meriden, CT, USA). The spectrum was collected in mirror-image mode over 1024 channels and folded about a 0-point of velocity defined by the spectrum of a thin foil of metallic iron collected simultaneously with that of the unknown sample. Analysis of the spectrum was carried out using a least-squares fitting routine based on a lorentzian peak shape for the absorption features.

## RESULTS

Despite the nutrient-limited nature of Roraima Tepui, throughout RSC there was evidence of significant microbial activity on the rock surfaces, primarily through the presence of observable microbial colonies (**Figure 1B**). Using a luminescence-based assay for ATP to serve as a proxy for the presence of microorganisms (Venkateswaran et al., 2003), we measured relative luminescent units (RLU) values as high as 34,062 from surfaces within the cave. These values suggest a high amount of microbial activity when compared to other (carbonate) cave systems (such caves generally range from 80 to 1,400 RLU; Johnston and Barton, unpublished results). To explore this microbial activity, we examined three sites within the cave: CP, RR, and LG, which were progressively further from the entrance (CP ∼55 m, RR ∼90 m, and LG ∼300 m, respectively; **Figure 1C**). Observable microbial colonies were generally associated with turbulent water in the cave and became patchier deeper into the cave system. We therefore decided to collect samples where specific microbial growth was not observed, which was more representative of the majority of surface sediments within the cave. Using the ATP assay, we obtained a range of 4,025–15,352 RLU from these surface sites (**Table 1**), which provides an approximation of surficial cell numbers (assuming that cell ATP levels average ∼1 × 10^-18^ M) ranging from 2.07 to 7.65 × 10^7^ cells/cm^2^ (**Table 1**; La Duc et al., 2007).

**Table 1:** Physiochemical parameters at the different sampling sites.

Site	ATP (RLU)	ATP calculated cell number (/cm^2^)	Cell counts (/cm^3^)	Temperature °C	Humidity	Stream pH	Dissolved Si (mg/L)
Surface	452	0.45 × 10^7^	–	–	–	6.875	5.0
CP	4,025	2.01 × 10^7^	Nd	11.8	98.2%	5.561	4.0
RR	15,352	7.65 × 10^7^	1.92 × 10^8^ (SD ± 10.4%)	11.6	99.6%	5.097	6.0
LG	8,933	4.45 × 10^7^	0.52 × 10^8^ (SD ± 12.1%)	12.5	99.9%	4.968	15.0

*nd, not done; SD, standard deviation*.

To correlate the observed ATP values with total number of microbial cells at each site, we attempted both direct cell counting and fluorescent *in situ* hybridization (FISH). While cell counting using the DNA stain SYBR Green I was possible, the autofluorescence and DNA binding properties of the quartz grains meant that FISH could not be effectively used to distinguish community composition. Nonetheless, cell counts at LG (∼0.52 × 10^8^ cells/cm^3^) and RR (∼1.92 × 10^8^ cells/cm^3^) correlated well with the surficial ATP values (the collected CP sample was destroyed during transport and no cell numbers were obtained) and confirmed our observation that the cave contained a high microbial cell number (Venkateswaran et al., 2003; Barton et al., 2005, 2007). While we did attempt cultivation studies to determine the number of colony forming units at each site, the cultured isolates shared no similarity with the dominant phylotypes identified by non-cultivation (DNA) techniques, suggesting that colony counting was in no way representative of the number of species growing *in situ* (data not shown).

### MICROBIAL COMMUNITY STRUCTURE

Extraction of DNA from orthoquartzite is extremely difficult due to the glass-like nature of the rock, which adsorbs DNA to its surface (Mao et al., 1994). Nonetheless, by blocking the quartz with a synthetic nucleotide polymer prior to extraction, we were able to obtain sufficient DNA to generate 16S ribosomal RNA gene sequence libraries for bacteria and archaea. The bacterial clone libraries contained 184, 52, and 43 clones for CP, RR, and LG; this diminishing number of clones at each site was due to the increasing difficulty in extracting DNA as sample sites progressed further into the cave. A total of 87 and 91 archaeal phylotypes were obtained for the CP and RR sites, respectively, while the LG sample was completely consumed in repeated attempts to obtain sufficient DNA for PCR amplification. The inability to obtain archaeal clones from the LG site is thus the result of DNA extraction problems, rather than the absence of these organisms.

**FIGURE 2:**
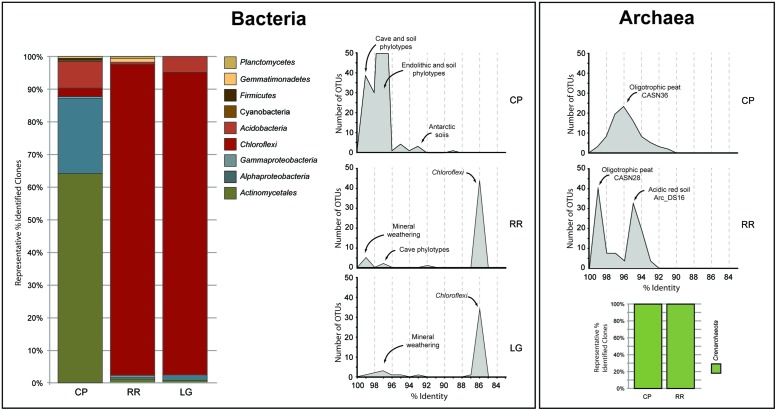
**Sequence analysis of identified phylotypes at each sample site within Roraima Sur Cave.** The bacterial and archaeal populations were analyzed separately and demonstrate the overall distribution of phyla identified at each site (bar charts). The distributions of phylotypes with BLAST analysis identity scores (%) to sequences in the Genbank database are shown, along with the major community composition or environmental source.

In order to obtain a general idea of community structure at each site we carried out a BLAST analysis (**Figure 2**). This analysis revealed that within the bacterial population there were distinct community differences between the near-entrance (CP) and deeper (RR and LG) sample sites. The CP bacterial population was dominated by the *Actinomycetales* and *Alphaproteobacteria*, along with a significant population of *Firmicutes* and *Acidobacteria* (**Figure 2**). The actinobacterial population at the CP site was itself dominated by members of the *Pseudonocardia*, while the *Alphaproteobacteria* were represented by a number of the nitrogen-fixing *Beijerinckiaceae* and *Methylocella* (Dedysh et al., 2004). The comparative BLAST of the remaining sequences revealed that many share identity with phylotypes also seen in mineral weathering horizons or geologic environments (caves, lava deposits, mines, and iron-manganese nodules). A single cyanobacterial clone was detected in the CP clone library; however, no *Cyanobacteria* were detected at any other site within the cave.

**FIGURE 3:**
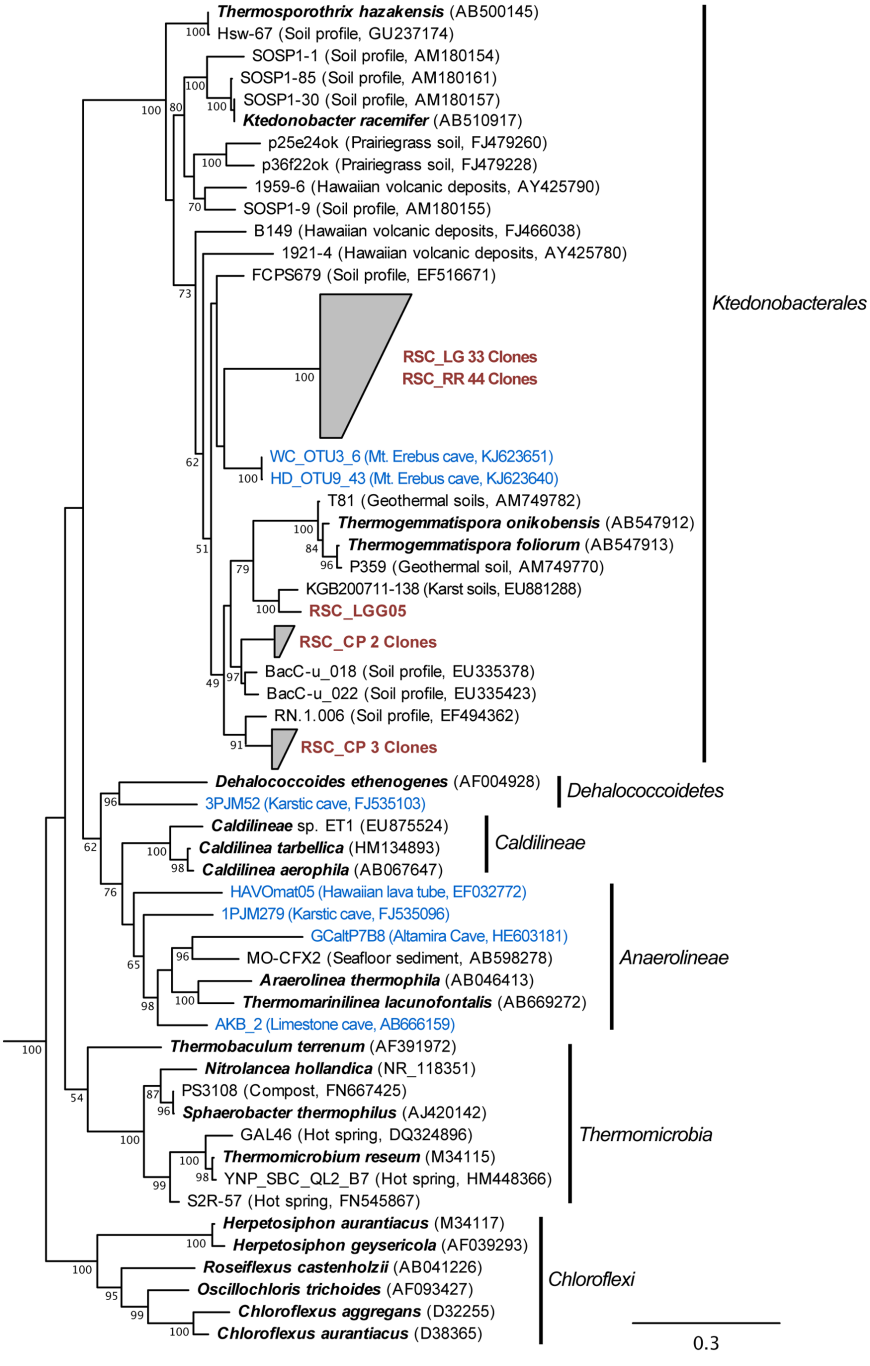
**Phylogenetic analysis of 16S rRNA gene sequences for the major Classes (Sub-phyla) within the *Chloroflexi,* along with representative phylotypes identified in other cave environments (blue) and this study (brown).** Cultured *Chloroflexi* isolates are shown in bold. The tree topology is based on a maximum likelihood analysis using RAxML and the evolutionary model CTR + G. The lowest scoring tree is shown, with branch support (percentage) of 1,000 bootstrap replicates shown. The scale bar represents the estimated number of replacements at each site. The bacterial 16S sequences for *Aquifex pyrophilus* (M83548) and *Hydrogenobacter thermophilus* (Z30214) were used as an outgroup.

The bacterial populations at RR and LG sites look remarkably similar, with the dominance of a phylotype showing a low level of sequence identity to the *Chloroflexi* (86%; **Figure 2**). Despite the dominance of the *Chloroflexi*, these sites did contain a minority population of both the *Actinobacteria* (4% at RR and 2% at LG) and *Alphaproteobacteria* (2% at RR and 5% at LG). The RR library also contained members of the *Gemmatimonadetes* and *Planctomycetes*, while LG contained a number of *Acidobacteria* (**Figure 2**); representatives of these phyla shared a higher degree of identity to organisms examined in other environments than representatives of the *Chloroflexi* (**Figure 2**). Given the uniqueness of the *Chloroflexi*, we wanted to determine whether they shared any homology with other *Chloroflexi* previously identified in caves (Barton, 2014). The resultant phylogeny (**Figure 3**) demonstrates that the RSC *Chloroflexi* group falls within the Class *Ktedonobacterales*, while the *Chloroflexi* that have been identified in past cave studies associate with the Classes *Dehalococcoidetes* and *Anaerolineae.* It is interesting to note that there is a distinct lineage within the *Chloroflexi* from RSC, with phylotypes from deeper in the cave (RR and LG) forming a unique clade with clones from a fumarole cave on Mount Erebus, Antarctica (**Figure 3**), while (apart from a single phylotype identified at the LG; RSC_LGG05) those found near the entrance at CP share an evolutionary history with soil-associated clades (**Figure 3**).

**FIGURE 4:**
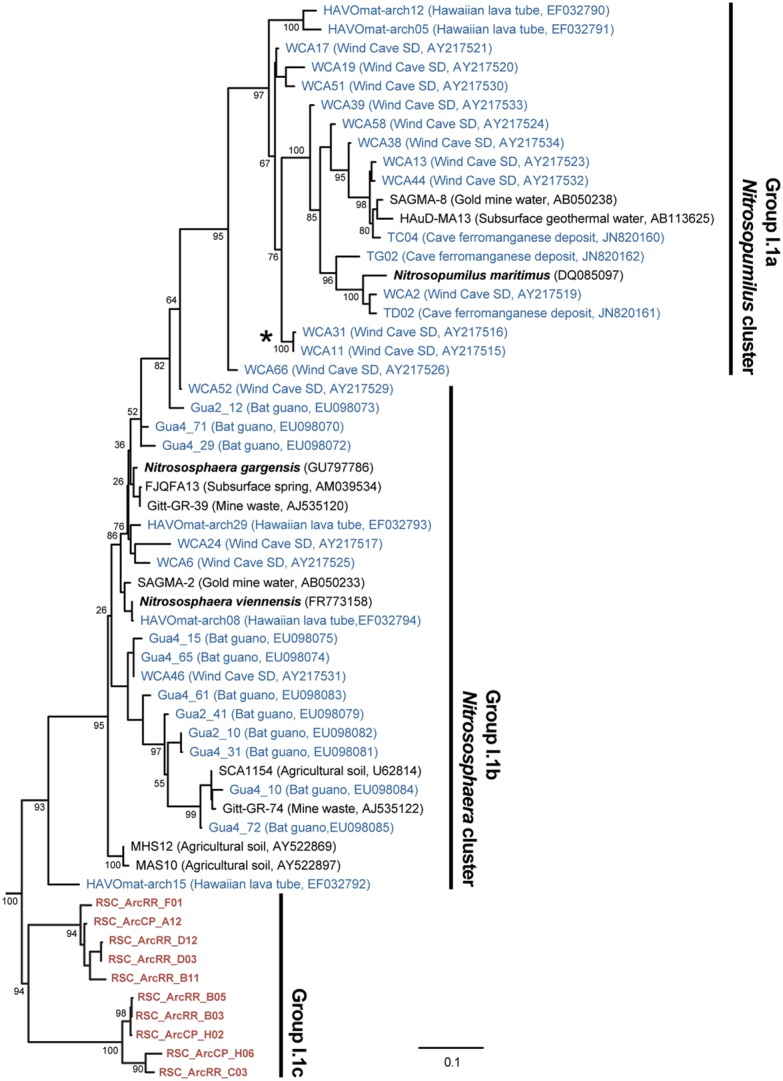
**Phylogenetic analysis of the *Thaumarchaeota* Group I (as defined by DeLong, 1992) 16S rRNA gene sequences, including representative phylotypes identified from other (carbonate) cave environments (blue) and this study (red).** Cultured *Thaumarchaeota* isolates are shown in bold. The clade indicated with the (*) represents the relative location of the *Crenarchaeota* identified from Lechuguilla Cave (Northup et al., 2003). The tree topology is based on a maximum likelihood analysis using RAxML and the evolutionary model CTR + G. The lowest scoring tree is shown, with branch support (percentage) of 1,000 bootstrap replicates shown. The scale bar represents the estimated number of replacements at each site. The archaeal 16S sequences for *Methanobacterium aarhusense* H2-LR (AY386124) and *Ferroplasma acidiphilum* MT1 (AF513710) were used as an outgroup.

**FIGURE 5:**
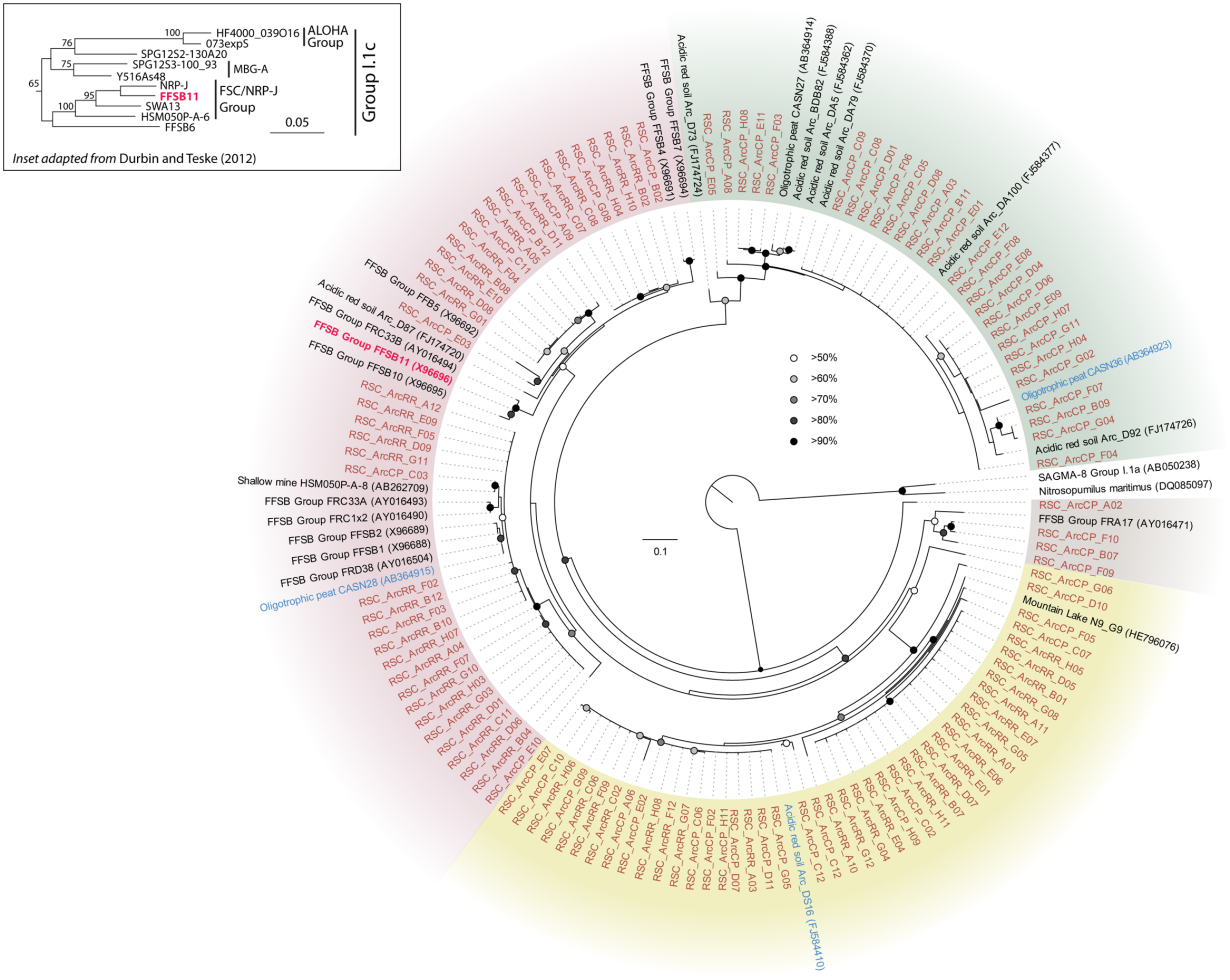
**Phylogenetic analysis of the *Thaumarchaeota* Group I.1c FSC/NRP-J Group (as defined by Durbin and Teske, 2012; inset) 16S rRNA gene sequences.** The sequences from this study are shown (orange) as well as BLAST identified sequences (blue), the FSSB11 sequence of the FSC/NRP-J group (red), and sequences in the FSC/NRP-J group as identified from the RDP, SILVA and Genbank databases. The tree topology is based on a maximum likelihood analysis using RAxML and the evolutionary model CTR + G. The lowest scoring tree is shown, with branch support (percentage) of 1,000 bootstrap represented by circles (as shown). The scale bar represents the estimated number of replacements at each site, while background coloring was used to highlight each of the putative clades. The *Thaumarchaeota* Group I.1a 16S sequences for *Nitrosopumilus maritimus* SCM1 (DQ085097) and the uncultured clone SAGMA-8 (AB050238) were used as an outgroup.

The BLAST analysis of the archaea at CP and RR indicated that all the phylotypes belonged to the *Crenarchaeota* in two distinct populations: the majority of phylotypes from the CP site shared sequence identity with an oligotrophic peat clone (CASN36; Akiyama et al., 2011), while the RR site was dominated by two phylotypes, one with similarity to the same peat study (CASN28) and the other from an acidic desert soil (Arc_DS16; Ying et al., 2010; Akiyama et al., 2011; **Figure 2**). As with the *Chloroflexi*, we carried out a phylogenetic analysis to relate the RSC archaeal clones to other *Crenarchaeota* populations previously identified in caves. Given the ambient conditions within the cave (**Table 1**), it was unsurprising that all of the identified phylotypes clustered with the mesophilic *Crenarchaeota* ammonia-oxidizing Class *Thaumarchaeota*; however, the RSC phylotypes formed a distinct cluster within the *Thaumarchaeota* Group I.1c (**Figure 4**). When we searched the RDP, SILVA, and Genbank databases for additional sequences with shared homology, the RSC archaeal clones clustered within a subgroup designated NRP-J by DeSantis and colleagues (McDonald et al., 2012), which has variously been classified as the MBG-A affiliated, FSC, and FFSB Group (Jurgens et al., 1997; Vetriani et al., 1999; Takai et al., 2001). Due to this uncertainty in the phylogeny of the *Thaumarchaeota*, we used the more robust framework of Durbin and Teske (2012) to determine the phylogenetic placement of our clones. The resultant phylogeny (**Figure 5**) demonstrated that the RSC archaeal phylotypes clustered within the FSC/NRP-J group in four distinct clades, three of which correlated well with the peaks observed in our initial BLAST analyses (**Figure 2**). All of the sequences used to determine the phylogeny of the FSC/NRP-J Group have been identified in acidic environments, including acidic (Arc_DS16) and humic soils (FRA27), oligotrophic peat (CASN28 and CASN36) and mines (HSM050P-A-8; Jurgens et al., 1997; Oline et al., 2006; Ying et al., 2010; Akiyama et al., 2011), suggesting that pH plays a major role in the archaeal community structure within the cave.

### GEOCHEMICAL ANALYSIS

**FIGURE 6:**
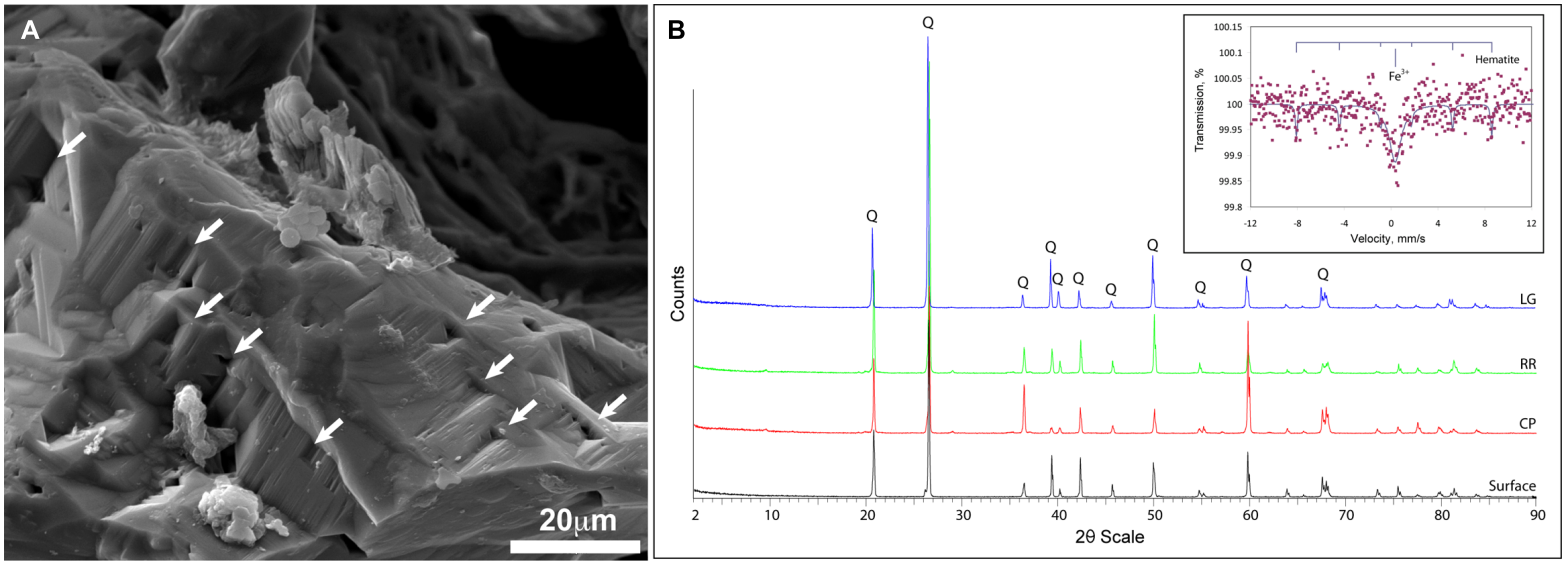
**Geochemical analyses of Roraima Sur Cave samples. (A)** SEM analysis of the quartz grains reveals the evidence of microbial activity and the etch-pits (indicated by arrows) characteristic of chemical dissolution. **(B)** XRD diffraction pattern of host rock material from outside of the cave (surface) and the sample sites, Cricket Pool (CP), Red River (RR), and Largo Grande (LG). Q indicates the presence of a recognized α-quartz peak; (*inset*) Mössbauer spectra of the LG site, with reference peaks for hematite.

Given the dramatic difference in diversity between CP and the deeper sites within the cave and the potential role of pH (RR and LG), we examined whether the geochemistry in RSC played a role in driving community structure. The physiochemical conditions in the cave were relative stable, with a temperature of 11.8°C at CP increasing to 12.5°C at LG, as the RH reached near-saturation at 99.9% (**Table 1**). Given the insolubility of the orthoquartzite, the stream provided the only entry of allochthonous nutrients into the cave, while presumably being responsible for the observed humidity. The measured pH of the stream did vary, dropping from 5.561 at CP to 4.968 at LG (**Table 1**). Gross examination of geologic hand-samples demonstrated that the orthoquartzite at each location had lost its rock-like structure and was turning into a sandy sediment, presumably through the dissolution of the silica cement (Martini, 2003). SEM analysis of the sediments confirmed this analysis, and revealed the presence of triangular etch pits within the quartz grains suggesting that chemical weathering of the mineral surface was occurring (**Figure 6A**; Turkington and Paradise, 2005). The degradation of the host rock into sandy sediment correlated with an increase in dissolved silica in the stream (**Table 1**), which could be caused by the production of a weak silicic acid (H_4_SiO_4_).

Total elemental and X-ray powder diffraction (XRD) analyses of the sediments confirms that SiO_2_ represents the predominant chemistry of the orthoquartzite in its mineral polymorph α-quartz (**Table 1**; **Figure 6B**). Other predominant elements within the rock include aluminum (1.96–3.00%), which is enriched in the cave when compared to the Tepui surface, and iron (0.08–0.12%), which is likely responsible for the pink color of the rock (**Figure 1**; **Table 2**). We identified trace phosphorous (0.02–0.05%), which has the potential to serve as a nutrient; potassium, strontium, calcium, barium, sodium and magnesium were below the 0.01% sensitivity of the instrument. In order to examine whether the observed iron could contribute to microbial metabolism, either through autotrophic or mixotrophic growth, we used Mossbauer spectroscopy. The obtained spectrum demonstrates a very low absorption effect and poor signal/noise ratio (**Figure 6B**) and confirms both the low level of available iron and its presence as hematite [Fe(III)]. Given the dominance of ammonia-oxidizing species within RSC, we also tested for the presence of nitrogenous compounds using a field-available assay. While we found no reactive nitrogenous compounds (${NH}_{4}^{+}$, ${NO}_{2}^{-}$, ${NO}_{3}^{-}$) from rocks on the surface of the Tepui, outside of the cave (**Table 2**), both nitrate and ammonia were detected at trace levels at all sample sites within the cave. Together these data suggest that while there are some differences between the geochemistry of the surface and the cave sites, there are no significant geochemistry differences to account for the observed changes in microbial community structure within the cave.

**Table 2:** Geochemistry of sample sites.

Sample site	Chemical Parameter							
	Si	Al	P	Fe	Ti	${NH}_{4}^{+}$	${NO}_{2}^{-}$	${NO}_{3}^{-}$
		
	%					mg/L			
Surface rock	99.40	0.45	0.02	0.07	0.07	*bdl*	*bdl*	*bdl*
CP	97.29	2.76	0.05	0.08	0.04	<0.25	*bdl*	<0.5
RR	95.99	3.00	0.04	0.12	0.07	<0.25	*bdl*	<0.5
LG	98.94	1.96	0.02	0.10	0.02	<0.25	*bdl*	<0.5

**bdl*, below detection limit*.

## DISCUSSION

**FIGURE 7:**
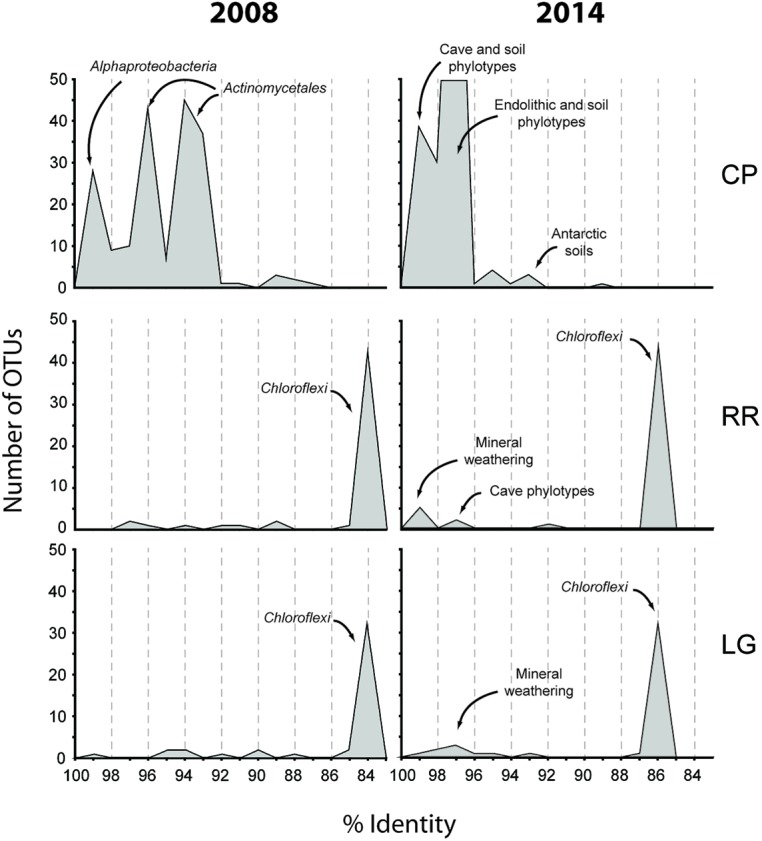
**Initial (2008) and subsequent re-analysis (2014) of Roraima Sur Cave phylotypes for this volume.** The distributions of phylotypes with BLAST analysis identity scores (%) to sequences in the Genbank database are shown, along with the major community composition or environmental source.

The Tepui Mountains of Venezuela are a remarkable environment that contains some of the longest and deepest quartzite caves in the world (Martini, 2003; Auler, 2004; Galan et al., 2004; Aubrecht et al., 2008, 2011). They are also among the most remote and inaccessible environments for research. Nonetheless, given the history of unusual fauna and flora found on these mountains (Im Thurn, 1887; Maguire, 1970; Steyermark, 1979), it was unsurprising that a unique microbial community was found within its caves; this uniqueness was evident when we first processed the data from this cave in 2008 (**Figure 7**). At the time the *Thaumarchaeota* had yet to be described, the cultivation of *Nitrosopumilus maritimus* still appeared to be novel, our knowledge of the contribution by ammonia-oxidizing archaea (AOA) in the global nitrogen cycle was still in its infancy, and the *Ktedonobacterales* had only recently been described from the type strain *Ktedonobacter racemifer* (Venter et al., 2004; Könneke et al., 2005; Cavaletti et al., 2006; Brochier-Armanet et al., 2008). Next-generation sequencing technologies remained limited and expensive, and unable to amplify low-biomass samples (<50 ng) without significant (and biased) amplification. We were therefore confronted with a 16S rRNA clone library that comprised of sequences with very little identity to characterized species within the Genbank database (**Figure 7**). Even the bacterial phylotypes within the CP site, which demonstrated the best identity to known sequences, left us with what appeared to be a soil-like community (**Figure 7**). Thus the original analysis of the RSC microbial populations provided very little functional information.

Since that time, more than 6.1 × 10^7^ sequences (representing a total of 3.3 × 10^9^ bases) have been added to the Genbank database (Genbank release notes^4^), including 16S rRNA sequences from newly explored geochemical environments (Connell and Staudigel, 2013). Collectively this knowledge increase produced a noticeable shift in the identity of our sequences to those within the Genbank database (**Figure 7**) and has improved our ability to determine an environmental physiology of the microbial community in RSC. For example, at the CP site, in addition to soil-associated species, our clones now demonstrate sequence identity to phylotypes from caves, mineral weathering surfaces, and endolithic environments (**Figure 7**), including heterotrophic, nitrogen-fixing species identified on the surfaces of carbonate caves impacted by the introduction of organic carbon (Stomeo et al., 2008). The presence of *Cyanobacteria* at CP may be indicative of the close proximity of this sample site to the entrance and colonization by surface species (Büdel, 1999); indeed the site is close enough to the cave entrance that, by peering around a corner, daylight can be seen (**Figure 1**). Alternatively, the presence of so many heterotrophic, nitrogen-fixing species at CP suggests that the microorganisms depend on surface-derived carbon for growth, while the ability to fix nitrogen may play a critical role in microbial subsistence. Taken together, these new data suggest that the CP community exists within a transitional zone, where the microbial community is still influenced by surface colonization or allochthonous carbon input, but demonstrates some endolithic-like adaptation to life on the silicate mineral surface.

As the sample sites extended further into the cave, there was a dramatic shift in bacterial community structure (**Figure 2**). The impermeable, orthoquartzite rock prevents allochthonous infiltration, and thus these sample sites (RR and LG) would have to depend on organic input directly from the cave stream or autotrophic activities. In support of the stream hypothesis, the absence of surface soils on the Tepuis means that rainwater rapidly accumulates dissolved organic matter (DOM) from plant detritus and humic material before flowing into the cave; the amount of humic material in the water flowing into the cave and off of the Tepuis can be so high that it gives the rivers in this region their famous tannic “black” color (Piccini and Mecchia, 2009). While equipment failure meant we were unable to measure DOM at the time of sampling, the water on Tepui mountains has an average dissolved DOM content of ∼19 mg/L (Gorbushina et al., 2001), which is much higher than the average measured DOM levels in carbonate caves (<0.5 mg/L; Barton, 2014). This comparatively high DOM flowing into the cave could certainly serve as the source of energy that drives the high levels of microbial activity observed and indeed, the most visible microbial activity seen was associated with turbulent water flow (**Figure 1**); however, if this were the case, it is unclear why the community deeper into the cave contains such a dominant population *Ktedonobacterales*, rather than the phyla seen at the near-entrance CP site that are more commonly associated with the breakdown of plant detritus (**Figures 2** and **3**; Hug et al., 2013).

The *Chloroflexi* represent a remarkably diverse group, with a phylogenetic range as broad as that of the *Proteobacteria* (Ley et al., 2006); yet out of the 20,702 16S rRNA sequences in the RDP database, there are only 187 cultured representatives (this compares with 96,507 cultured isolates for the *Proteobacteria*). This makes it very difficult to estimate the metabolic function for uncultured species within the environment, particularly in this study, when the closest cultured representative (*Thermogemmatispora onikobensis*) only has 84% sequence identity at the 16S rRNA gene level (Cole et al., 2005). Nonetheless, the *Chloroflexi*, and in particular the *Ktedonobacterales*, have a recognized role as heterotrophic oligotrophs in soils, including the ability to survive on more recalcitrant plant polymers (Yabe et al., 2010, 2011; Hug et al., 2013; King and King, 2014). Their presence in oligotrophic environments, including caves, confirms this adaptation to growth under nutrient limitation (Engel et al., 2010; Hug et al., 2013; Barton, 2014). The difference in *Ktedonobacterales* phylotypes between the near-entrance CP and deeper cave sites, along with their dominance at RR and LG, suggest that there are specific selective pressures deeper within the cave for these organisms. The unique clade formed by the RSC phylotypes and those from a mesophilic fumarole cave on Mount Erebus, Antarctica (**Figure 3**) has the potential to tell us more about such potential selective pressures (Yabe et al., 2011).

The RR and LG sites are dominated by representatives of the *Ktedonobacterales* growing on a silica-rich substrate in the form of quartz (**Figure 6**). The fumarole caves, which form at the contact between the ice sheet and a floor of phonolitic lava, similarly contain *Ktedonobacterales* growing in a lithosoil comprised of silica-rich montmorillonite and kaolinite, with a pH of 4.1–5.8 (Ugolini, 1965; Hudson and Daniel, 1988; Connell and Staudigel, 2013). In contrast, the *Dehalococcoidetes* and *Anaerolineae* dominate the *Chloroflexi* in well-buffered (pH 8.0–8.3) limestone caves, as well as and Hawaiian lava tube caves (**Figure 4**), where the basaltic lava (<20% quartz) is much less susceptible to chemical weathering (Porder et al., 2007). Together, these data suggest that the selection pressure for the *Ktedonobacterales* may be related to either the high levels of silica, and a consequence of the selective pressure of the Si^4+^ ion, or the result of the poor buffering and the surface acidity of quartz and phyllosilicates (Porder et al., 2007). Given the observation of the *Thaumarchaeota* Group I.1c in RSC, it is likely that pH may be the predominant driver of community structure within the microbial ecosystem.

DeLong (1992) first identified a group of mesophilic, marine *Crenarchaeota* that metagenomic analyses suggested contained an ammonia monooxygenase (*amo*A) gene and ammonia-oxidizing activity (Venter et al., 2004). Upon the discovery of related sequences in the soil, these *Crenarchaeota* were subsequently reclassified into Groups I.1a, I.1b, and I.1c: Group I.1a being associated with marine and freshwater environments, Group I.1b with soil and subsurface environments, and Group I.1c with forest soils (Jurgens et al., 1997; Ochsenreiter et al., 2003). The significance of these *Crenarchaeotal* populations within the environment was demonstrated by cultivation of the ammonia-oxidizing *Nitrosopumilus maritimus*, which confirmed their ammonia-oxidizing potential (Könneke et al., 2005). Since that time, these mesophilic AOA have been classified into a new phylum, the *Thaumarchaeota*, which are now recognized to be important, if not the dominant, players in global nitrification (Brochier-Armanet et al., 2008; Monteiro et al., 2014).

As more members of the *Thaumarchaeota* were identified within the environment, there seemed to be an association of Group I.1c with acidic environments, even though the ammonia ion is protonated to its unfavorable ammonium form at low pH. Nonetheless, both pH and nutrient conditions appear to be important drivers of niche-differentiation within the *Thaumarchaeota* (Lehtovirta et al., 2009; Martens-Habbena et al., 2009; Gubry-Rangin et al., 2011; Auguet and Casamayor, 2013). The lack of sufficient ammonia for ammonia-oxidation under low pH was solved with the cultivation of the acidic AOA *Nitrososphaera viennensis*, which uses urea for growth (Tourna et al., 2011). This urea, which appears to play a critical role in ammonia-oxidation under acidic conditions, is degraded by intracellular ureases to release the necessary ammonia for growth (Lu et al., 2012; Lu and Jia, 2013). Thus, the low pH within RSC may therefore explain both the presence of the *Thaumarchaeota* Group I.1c and the *Ktedonobacterales*, which encode ureases and can use nitrite and nitrate (Costello and Schmidt, 2006; Hanada and Pierson, 2006; Wu et al., 2009; Sorokin et al., 2012). If ammonia and/or urea are driving community structure within RSC, the question remains as to its source. The answer may require us to re-examine the stream entering the cave.

Cave microbiology is a relatively young field, although there has been a dramatic increase in recent years in both the number of research groups and resultant publications (Lee et al., 2012). Yet much of this work has centered on traditional limestone (karst) caves. Very little microbial exploration has taken place in pseudokarst – caves found in rock other than limestone (Halliday, 2007). Such environments include lava caves, ice, glacier and fumarole caves, talus caves, iron caves, littoral sea caves, and sandstone caves (Hudson and Daniel, 1988; Soo et al., 2009; Northup et al., 2011; Connell and Staudigel, 2013; Hathaway et al., 2014). Yet such caves contribute significantly to our understanding of the geochemical environments and subsurface ecosystems that can be studied on Earth (Herbold et al., 2014). The difficulty in accessibility, unforgiving sampling environment, and difficulty in obtaining DNA from these samples means that this initial work has only allowed us a snapshot of the microbial diversity found within the tepui sandstone caves. With advanced technologies in low-biomass next-generation sequencing, metagenomic approaches, and the potential to culture the *Thaumarchaeota*, we hope that further study will allow us to better understand both the active physiologies and the drivers of microbial selection within these unusual microbial ecosystems.

## AUTHOR CONTRIBUTIONS

All authors contributed to the conception or design of the work, fieldwork, data analysis and interpretation. The manuscript was prepared by Hazel A. Barton, with assistance from Juan G. Giarrizzo and Charles E. Robertson.

## Conflict of Interest Statement

The authors declare that the research was conducted in the absence of any commercial or financial relationships that could be construed as a potential conflict of interest.

## Footnotes

1 http://greengenes.lbl.gov

2 http://www.ncbi.nlm.nih.gov/

3 http://tree.bio.ed.ac.uk/software/figtree/

4 ftp.ncbi.nih.gov/genbank.gbrel.txt

## References

[B1] Akiyama M. Shimizu S. Sakai T. Ioka S. Ishijima Y. Naganuma T. (2011). Spatiotemporal variations in the abundances of the prokaryotic rRNA genes, *pmoA*, and *mcrA* in the deep layers of a peat bog in Sarobetsu-genya wetland, Japan. *Limnology* 12 1–9 10.1007/s10201-010-0315-3

[B2] Allen B. L. Hajek B. F. (1989). “Mineral occurrences in soil environments,” in *Minerals in Soil Environments* eds Dixon J. B. Weed S. B. (Madison, WI: Science Society of America), 199–264.

[B3] Angert E. R. Northup D. E. Reysenbach A.-L. Peek A. S. Goebel B. M. Pace N. R. (1998). Molecular phylogenetic analysis of a bacterial community in Sulphur River, Parker Cave, Kentucky. *Am. Mineral.* 83 1583–1592.

[B4] Aubrecht R. Lánczos T. Gregor M. Schlögl J. Šmída B. Liščák P. (2011). Sandstones caves on Venezuela tepuis: return to pseudokarst? *Geomorphology* 132 351–365 10.1016/j.geomorph.2011.05.023

[B5] Aubrecht R. Lanczos T. Smida B. Brewer-Carias C. Mayoral F. Schlogl J. (2008). Venezuelan sandstone caves: a new view on their genesis, hydrogeology and speleothems. *Geologia Croatia* 61 345–362.

[B6] Auguet J. C. Casamayor E. O. (2013). Partitioning of Thaumarchaeota populations along environmental gradients in high mountain lakes. *FEMS Microbiol. Ecol.* 84 154–164 10.1111/1574-6941.12047 23176712

[B7] Auler A. (2004). “Quartzite caves of South America,” in *Encyclopedia of Cave and Karst Science* ed. Gunn J. (New York: Taylor and Francis), 611–613.

[B8] Banks E. D. Taylor N. M. Gulley J. Lubbers B. R. Giarrizo J. G. Bullen H. A. (2010). Bacterial calcium carbonate precipitation in cave environments: a function of calcium homeostasis. *Geomicrobiol. J.* 27 444–454 10.1080/01490450903485136

[B9] Barton H. A. (2013). “Biospeleogenesis,” in *Treatise on Geomorphology* ed. Shroder J. (Amsterdam: Academic Press), 6000.

[B10] Barton H. A. (2014). “Starving artists: bacterial oligotrophic heterotrophy in caves,” in *Life in Extreme Environments: Microbial Life of Cave Systems* ed. Wagner D. (Berlin: DeGruyter).

[B11] Barton H. A. Pemberton A. Millette J. (2005). “Comparative study of oligotrophic bacterial species cultivated from Jack Bradley Cave, Kentucky,” in *Proceedings of the 14th International Congress of Speleology* Athens.

[B12] Barton H. A. Taylor M. R. Pace N. R. (2004). Molecular phylogenetic analysis of a bacterial community in an oligotrophic cave environment. *J. Geomicrobiol.* 21 11–20 10.1080/01490450490253428

[B13] Barton H. A. Taylor N. M. Kreate M. Springer A. J. Oehrle S. A. Bertog J. L. (2007). The impact of host rock geochemistry on bacterial community structure in oligotrophic cave environments. *Int. J. Speleol.* 36 93–104 10.5038/1827-806X.36.2.5

[B14] Barton H. A. Taylor N. M. Lubbers B. R. Pemberton A. C. (2006). DNA extraction from low-biomass carbonate rock: an improved method with reduced contamination and the low-biomass contaminant database. *J. Microbiol. Methods* 66 21–31 10.1016/j.mimet.2005.10.005 16305811

[B15] Bhullar K. Waglechner N. Pawlowski A. Koteva K. Banks E. D. Johnston M. D. (2012). Antibiotic resistance is prevalent in an isolated cave microbiome. *PLoS ONE* 7:e34953 10.1371/journal.pone.0034953 PMC3324550 22509370

[B16] Briceno H. Schubert C. Paolini J. (1990). Table mountain geology and superficial geochemistry: Chimanta Masif, Venezuelan Guyana Shield. *J. South Am. Earth Sci.* 3 179–194 10.1016/0895-9811(90)90002-I

[B17] Brochier-Armanet C. Boussau B. Gribaldo S. Forterre P. (2008). Mesophilic Crenarchaeota: proposal for a third archaeal phylum, the Thaumarchaeota. *Nat. Rev. Microbiol.* 6 245–252 10.1038/nrmicro1852 18274537

[B18] Büdel B. (1999). Ecology and diversity of rock-inhabiting cyanobacteria in tropical regions. *Eur. J. Phycol.* 34 361–370 10.1080/09670269910001736422

[B19] Cavaletti L. Monciardini P. Bamonte R. Schumann P. Rohde M. Sosio M. (2006). New lineage of filamentous, spore-forming, gram-positive bacteria from soil. *Appl. Environ. Microbiol.* 72 4360–4369 10.1128/aem.00132-06 16751552 PMC1489649

[B20] Chelius M. K. Moore J. C. (2004). Molecular phylogenetic analysis of archaea and bacteria in wind cave, South Dakota. *Geomicrobiol. J.* 21 123–134 10.1080/01490450490266389

[B21] Cole J. R. Chai B. Farris R. J. Wang Q. Kulam S. A. Mcgarrell D. M. (2005). The Ribosomal Database Project (RDP-II): sequences and tools for high-throughput rRNA analysis. *Nucleic Acids Res.* 33 D294–D296 10.1093/nar/gki038 15608200 PMC539992

[B22] Connell L. Staudigel H. (2013). Fungal diversity in a Dark Oligotrophic Volcanic Ecosystem (DOVE) on Mount Erebus, Antarctica. *Biol. (Basel)* 2 798–809 10.3390/biology2020798 PMC3960884 24832809

[B23] Costello E. K. Schmidt S. K. (2006). Microbial diversity in alpine tundra wet meadow soil: novel Chloroflexi from a cold, water-saturated environment. *Environ. Microbiol.* 8 1471–1486 10.1111/j.1462-2920.2006.01041.x 16872409

[B24] Cuezva S. Fernandez-Cortes A. Porca E. Pašic L. Jurado V. Hernandez-Marine M. (2012). The biogeochemical role of Actinobacteria in Altamira Cave, Spain. *FEMS Microbiol. Ecol.* 81 281–290 10.1111/j.1574-6941.2012.01391.x 22500975

[B25] Dedysh S. N. Ricke P. Liesack W. (2004). NifH and NifD phylogenies: an evolutionary basis for understanding nitrogen fixation capabilities of methanotrophic bacteria. *Microbiology* 150 1301–1313 10.1099/mic.0.26585-0 15133093

[B26] DeLong E. F. (1992). Archaea in coastal marine environments. *Proc. Natl. Acad. Sci. U.S.A.* 89 5685–5689 10.1073/pnas.89.12.5685 1608980 PMC49357

[B27] Durbin A. M. Teske A. (2012). Archaea in organic-lean and organic-rich marine subsurface sediments: an environmental gradient reflected in distinct phylogenetic lineages. *Front. Microbiol.* 3:168 10.3389/fmicb.2012.00168 PMC3364523 22666218

[B28] Engel A. S. Meisinger D. B. Porter M. L. Payn R. A. Schmid M. Stern L. A. (2010). Linking phylogenetic and functional diversity to nutrient spiraling in microbial mats from Lower Kane Cave (USA). *ISME J.* 4 98–110 10.1038/ismej.2009.91 19675595

[B29] Galan C. Herrera F. F. Carreno R. Perez M. A. (2004). Roraima Sur System, Venezuela: 10.8 Km, World’s longest quartzite cave. *Bol. Soc. Venezolana Espel.* 38 53–60.

[B30] Gorbushina A. A. Boettcher M. Brumsack H.-J. Krumbein W. E. Vendrell-Saz M. (2001). Biogenic forsterite and opal as a product of biodeterioration and lichen stromatolite formation in table mountain systems (Tepuis) of Venezuela. *Geomicrobiology J.* 18 117–132 10.1080/01490450151079851

[B31] Groth I. Schumann P. Laiz L. Moral-Sanchez S. Canaveras J. C. Saiz-Jimenez C. (2001). Geomicrobiological study of the Grotta dei Cervi, Porto Badisco, Italy. *Geomicrobiol. J.* 18 241–258 10.1080/01490450152467778

[B32] Gubry-Rangin C. Hai B. Quince C. Engel M. Thomson B. C. James P. (2011). Niche specialization of terrestrial archaeal ammonia oxidizers. *Proc. Natl. Acad. Sci. U.S.A.* 108 21206–21211 10.1073/pnas.1109000108 22158986 PMC3248517

[B33] Hales B. A. Edwards C. Ritchie D. A. Hall G. Pickup R. W. Saunders J. R. (1996). Isolation and identification of methanogen-specific DNA from blanket bog peat by PCR amplification and sequence analysis. *Appl. Environ. Microbiol.* 69 74–83. 10.1128/aem.62.2.668-675.1996 PMC167834 8593069

[B34] Halliday W. R. (2007). Pseudokarst in the 21st century. *J. Cave Karst Stud.* 69 103–113.

[B35] Hanada S. Pierson B. K. (2006). “The family Chloroflexaceae” in *The Prokaryotes* eds Falkow S. Rosenberg E. Schleifer K. H. Stackebrandt E. Dworkin M. (New York: Springer), 815–842 10.1007/0-387-30747-8_33

[B36] Hathaway J. J. Sinsabaugh R. L. De Lurdes M. Dapkevicius N. E. Northup D. E. (2014). Diversity of ammonia oxidation (amoA) and nitrogen fixation (nifH) genes in lava caves of Terceira, Azores, Portugal. *Geomicrobiol. J.* 31 221–235 10.1080/01490451.2012.752424 PMC4711379 26778867

[B37] Herbold C. W. Lee C. K. Mcdonald I. R. Cary S. C. (2014). Evidence of global-scale aeolian dispersal and endemism in isolated geothermal microbial communities of Antarctica. *Nat. Commun.* 5 3875 10.1038/ncomms4875 24846491

[B38] Hudson J. A. Daniel R. M. (1988). Enumeration of thermophilic heterotrophs in geothermally heated soils from Mount Erebus, Ross Island, Antarctica. *Appl. Environ. Microbiol.* 54 622–624. 16347573 10.1128/aem.54.2.622-624.1988 PMC202510

[B39] Hug L. A. Castelle C. J. Wrighton K. C. Thomas B. C. Sharon I. Frischkorn K. R. (2013). Community genomic analyses constrain the distribution of metabolic traits across the Chloroflexi phylum and indicate roles in sediment carbon cycling. *Microbiome* 1 22 10.1186/2049-2618-1-22 PMC3971608 24450983

[B40] Im Thurn E. F. (1887). The botany of the Roraima expedition of 1884 (communicated by Sir J. D. Hooker). *Trans. Linn. Soc. Lond.* 2 249–300.

[B41] Jurgens G. Lindstrom K. Saano A. (1997). Novel group within the kingdom Crenarchaeota from boreal forest soil. *Appl. Environ. Microbiol.* 63 803–805. 9023962 10.1128/aem.63.2.803-805.1997 PMC168374

[B42] King C. E. King G. M. (2014). Description of *Thermogemmatispora carboxidivorans* sp. nov., a carbon-monoxide-oxidizing member of the class Ktedonobacteria isolated from a geothermally heated biofilm, and analysis of carbon monoxide oxidation by members of the class Ktedonobacteria. *Int. J. Syst. Evol. Microbiol.* 64 1244–1251 10.1099/ijs.0.059675-0 24425739

[B43] Klimchouk A. B. (2007). *Hypogean Speleogenesis: Hydrogeological and Morphological Perspective Carlsbad, NM*. Carlsbad, NM: National Cave and Karst Research Institute.

[B44] Klimchouk A. B. Ford D. C. Palmer A. N. Dreybrodt W. (2000). *Speleogenesis: Evolution of Karstic Aquifers*. Huntsville, AL: National Speleological Society.

[B45] Knudson H. W. Juday C. Meloche V. W. (1940). Silicomolybdate method for silica. *Ind. Eng. Chem. Anal. Ed.* 12 270–273 10.1021/ac50145a008

[B46] Könneke M. Bernhard A. E. De La Torre J. R. Walker C. B. Waterbury J. B. Stahl D. A. (2005). Isolation of an autotrophic ammonia-oxidizing marine archaeon. *Nature* 437 543–546 10.1038/nature03911 16177789

[B47] La Duc M. T. Dekas A. Osman S. Moissl C. Newcombe D. Venkateswaran K. (2007). Isolation and characterization of bacteria capable of tolerating the extreme conditions of clean room environments. *Appl. Environ. Microbiol.* 73 2600–2611 10.1128/aem.03007-06 17308177 PMC1855582

[B48] Larkin M. A. Blackshields G. Brown N. P. Chenna R. McGettigan P. A. McWilliam H. (2007). Clustal W and Clustal X version 2.0. *Bioinformatics* 23 2947–2948 10.1093/bioinformatics/btm404 17846036

[B49] Lee N. M. Meisinger D. B. Aubrecht R. Kovačik L. Saiz-Jimenez C. Baskar S. (2012). “Caves and karst environments,” in *Life at Extremes: Environments, Organisms and Strategies For Survival* ed. Bell E. M. (Egham: CAB International), 320–344.

[B50] Lehtovirta L. E. Prosser J. I. Nicol G. W. (2009). Soil pH regulates the abundance and diversity of Group 1.1c Crenarchaeota. *FEMS Microbiol. Ecol.* 70 367–376 10.1111/j.1574-6941.2009.00748.x 19732147

[B51] Ley R. E. Harris J. K. Wilcox J. Spear J. R. Miller S. R. Bebout B. M. (2006). Unexpected diversity and complexity of the Guerrero Negro hypersaline microbial mat. *Appl. Environ. Microbiol.* 72 3685–3695 10.1128/AEM.72.5.3685-3695.2006 16672518 PMC1472358

[B52] Lu L. Han W. Zhang J. Wu Y. Wang B. Lin X. (2012). Nitrification of archaeal ammonia oxidizers in acid soils is supported by hydrolysis of urea. *ISME J.* 6 1978–1984 10.1038/ismej.2012.45 22592820 PMC3446803

[B53] Lu L. Jia Z. (2013). Urease gene-containing Archaea dominate autotrophic ammonia oxidation in two acid soils. *Environ. Microbiol.* 15 1795–1809 10.1111/1462-2920.12071 23298189

[B54] Ludwig W. Strunk O. Westram R. Richter L. Meier H. Yadhukumar (2004). ARB: a software environment for sequence data. *Nucleic Acids Res.* 32 1363–1371 10.1093/nar/gkh293 14985472 PMC390282

[B55] Macalady J. L. Dattagupta S. Schaperdoth I. Jones D. S. Druschel G. K. Eastman D. (2008). Niche differentiation among sulfur-oxidizing bacterial populations in cave waters. *ISME J.* 2 590–601 10.1038/ismej.2008.25 18356823

[B56] Maguire B. (1970). On the flora of the Guayana Highland. *Biotropica* 2 85–100 10.2307/2989766

[B57] Mao Y. Daniel L. N. Whittaker N. Saffiotti U. (1994). DNA binding to crystalline silica characterized by Fourier-transform infrared spectroscopy. *Environ. Health Perspect.* 102(Suppl. 10), 165–171 10.1289/ehp.94102s10165 7705292 PMC1566983

[B58] Martens-Habbena W. Berube P. M. Urakawa H. De La Torre J. R. Stahl D. A. (2009). Ammonia oxidation kinetics determine niche separation of nitrifying Archaea and Bacteria. *Nature* 461 976–979 10.1038/nature08465 19794413

[B59] Martini J. E. J. (2003). “Silcate Karst,” in *Encyclopedia of Cave and Karst Science* ed. Gunn J. (New York: Routledge), 649–653.

[B60] McDonald D. Price M. N. Goodrich J. Nawrocki E. P. Desantis T. Z. Probst A. (2012). An improved Greengenes taxonomy with explicit ranks for ecological and evolutionary analyses of bacteria and archaea. *ISME J.* 6 610–618 10.1038/ismej.2011.139 22134646 PMC3280142

[B61] Michelangeli F. A. (2000). Species composition and species-area relationships in vegetation isolates on the summit of a sandstone mountain in southern Venezuela. *J. Trop. Ecol.* 16 69–82 10.1017/S0266467400001279

[B62] Miller M. A. Pfeiffer W. Schwartz T. (2010). “Creating the CIPRES science gateway for inference of large phylogenetic trees,” in *Gateway Computing Environments Workshop GCE*, 1–8 10.1109/GCE.2010.5676129

[B63] Monteiro M. Seneca J. Magalhaes C. (2014). The history of aerobic ammonia oxidizers: from the first discoveries to today. *J. Microbiol.* 52 537–547 10.1007/s12275-014-4114-0 24972807

[B64] Northup D. E. Barnes S. M. Yu L. E. Spilde M. N. Schelble R. T. Dano K. E. (2003). Diverse microbial communitiens inhabiting ferromanganese deposits in Lechuguilla and Spider Caves. *Environ. Microbiol.* 5 1071–1086 10.1046/j.1462-2920.2003.00500.x 14641587

[B65] Northup D. E. Melim L. A. Spilde M. N. Hathaway J. J. Garcia M. G. Moya M. (2011). Lava cave microbial communities within mats and secondary mineral deposits: implications for life detection on other planets. *Astrobiology* 11 601–618 10.1089/ast.2010.0562 21879833 PMC3176350

[B66] Ochsenreiter T. Selezi D. Quaiser A. Bonch-Osmolovskaya L. Schleper C. (2003). Diversity and abundance of Crenarchaeota in terrestrial habitats studied by 16S RNA surveys and real time PCR. *Environ. Microbiol.* 5 787–797 10.1046/j.1462-2920.2003.00476.x 12919414

[B67] Oline D. K. Schmidt S. K. Grant M. C. (2006). Biogeography and landscape-scale diversity of the dominant Crenarchaeota of soil. *Microb. Ecol.* 52 480–490 10.1007/s00248-006-9101-5 16909343

[B68] Ortiz M. Legatzki A. Neilson J. W. Fryslie B. Nelson W. M. Wing R. A. (2014). Making a living while starving in the dark: metagenomic insights into the energy dynamics of a carbonate cave. *ISME J.* 8 478–491 10.1038/ismej.2013.159 24030597 PMC3906820

[B69] Palmer A. N. Palmer M. V. (2000). Hydrochemical interpretation of Cave Patterns in the Guadalupe Mountains, New Mexico. *J. Cave Karst Stud.* 62 91–108.

[B70] Piccini L. Mecchia M. (2009). Solution weathering rate and origin of karst landforms and caves in the quartzite of Auyan-tepui (Gran Sabana, Venezuela). *Geomorphology* 106 15–25 10.1016/j.geomorph.2008.09.019

[B71] Porder S. Hilley G. E. Chadwick O. A. (2007). Chemical weathering, mass loss, and dust inputs across a climate by time matrix in the Hawaiian Islands. *Earth Planet. Sci. Lett.* 258 414–427 10.1016/j.epsl.2007.03.047

[B72] Quast C. Pruesse E. Yilmaz P. Gerken J. Schweer T. Yarza P. (2013). The SILVA ribosomal RNA gene database project: improved data processing and web-based tools. *Nucleic Acids Res.* 41 D590–D596 10.1093/nar/gks1219 23193283 PMC3531112

[B73] Santos J. O. S. Potter P. E. Reis N. J. Hartmann L. A. Fletcher I. R. Mcnaughton N. J. (2003). Age, source, and regional stratigraphy of the Roraima Supergroup and Roraima-like outliers in northern South America based on U-Pb geochronology. *GSA Bull.* 115 331–348 10.1130/0016-7606(2003)115<0331:ASARSO>2.0.CO;2

[B74] Sarbu S. M. Kane T. C. Kinkle B. K. (1996). A chemoautotrophically based cave ecosystem. *Science* 272 1953–1955 10.1126/science.272.5270.1953 8662497

[B75] Smida B. Brewer-Carias C. Audy M. Mayoral F. Vlcek L. Aubrecht R. (2008). “The longest quartzite caves of the world: Cueva Ojos de Cristal (16.1 km) and Cueva Charles Brewer (4.8 km) and other giant caves on Venezuela table-mountains tepuy Roraima and Chimanta discovered by our 7 expeditions in 2002–2007,” in *Proceedings of the fourth European Spelelogical Conference.* (Vercors: Federation Francaise de Speleologie), 239–243.

[B76] Soo R. M. Wood S. A. Grzymski J. J. Mcdonald I. R. Cary S. C. (2009). Microbial biodiversity of thermophilic communities in hot mineral soils of Tramway Ridge, Mount Erebus, Antarctica. *Environ. Microbiol.* 11 715–728 10.1111/j.1462-2920.2009.01859.x 19278453

[B77] Sorokin D. Y. Lucker S. Vejmelkova D. Kostrikina N. A. K‘rebezem R. Rijpstra W. I. C. (2012). Nitrification expanded: discovery, physiology and genomics of a nitrite-oxidizing bacterium from the phylum Chloroflexi. *ISME J.* 6 2245–2256 10.1038/ismej.2012.70 22763649 PMC3504966

[B78] Spear J. R. Barton H. A. Roberts J. K. Francis C. A. Pace N. R. (2007). Microbial community biofabrics in a Geothermal Mine Adit. *Appl. Environ. Microbiol.* 73 6172–6180 10.1128/AEM.00393-07 17693567 PMC2075011

[B79] Spear J. R. Walker J. J. McCollom T. M. Pace N. R. (2005). Hydrogen and bioenergetics in the Yellowstone geothermal ecosystem. *Proc. Natl. Acad. Sci. U.S.A.* 102 2555–2560 10.1073/pnas.0409574102 15671178 PMC548998

[B80] Stamatakis A. Hoover P. Rougemont J. (2008). A rapid Bootstrap algorithm for the RAxML web servers. *Syst. Biol.* 57 758–771 10.1080/10635150802429642 18853362

[B81] Steyermark J. A. (1979). Flora of the Guyana Highland: Endemicity of the generic flora of the summits of the Venezuela Tepuis. *Taxon* 28 45–54 10.2307/1219557

[B82] Stomeo F. Portillo M. C. Gonzalez J. M. Laiz L. Saiz-Jimenez C. (2008). *Pseudonocardia* in white colonizations in two caves with Paleolithic paintings. *Int. Biodeterior. Biodegradation* 62 483–486 10.1016/j.ibiod.2007.12.011

[B83] Takai K. Moser D. P. Deflaun M. Onstott T. C. Fredrickson J. K. (2001). Archaeal diversity in waters from deep South African gold mines. *Appl. Environ. Microbiol.* 67 5750–5760 10.1128/aem.67.21.5750-5760.2001 11722932 PMC93369

[B84] Tetu S. G. Breakwell K. Elbourne L. D. Holmes A. J. Gillings M. R. Paulsen I. T. (2013). Life in the dark: metagenomic evidence that a microbial slime community is driven by inorganic nitrogen metabolism. *ISME J.* 7 1227–1236 10.1038/ismej.2013.14 23426011 PMC3660674

[B85] Tourna M. Stieglmeier M. Spang A. Konneke M. Schintlmeister A. Urich T. (2011). *Nitrososphaera viennensis*, an ammonia oxidizing archaeon from soil. *Proc. Natl. Acad. Sci. U.S.A.* 108 8420–8425 10.1073/pnas.1013488108 21525411 PMC3100973

[B86] Turkington A. V. Paradise T. R. (2005). Sandstone weathering: a century of research and innovation. *Geomorphology* 67 229–253 10.1016/j.geomorph.2004.09.028

[B87] Ugolini F. C. (1965). Soils of Mount Erebus, Antarctica. *N. Z. J. Geol. Geophys.* 10 431–442 10.1080/00288306.1967.10426747

[B88] Venkateswaran K. Hattori N. La Duc M. T. Kern R. (2003). ATP as a biomarker of viable microorganisms in clean-room facilities. *J. Microbiol. Methods* 52 367–377 10.1016/S0167-7012(02)00192-6 12531506

[B89] Venter J. C. Remington K. Heidelberg J. F. Halpern A. L. Rusch D. Eisen J. A. (2004). Environmental genome shotgun sequencing of the Sargasso Sea. *Science* 304 66–74 10.1126/science.1093857 15001713

[B90] Vetriani C. Jannasch H. W. Macgregor B. J. Stahl D. A. Reysenbach A. L. (1999). Population structure and phylogenetic characterization of marine benthic Archaea in deep-sea sediments. *Appl. Environ. Microbiol.* 65 4375–4384. 10.1128/aem.65.10.4375-4384.1999 PMC91581 10508063

[B91] Wray R. A. L. (1997). A global review of solutional weathering forms on quartz sandstones. *Earth Sci. Rev.* 42 137–160 10.1016/S0012-8252(96)00056-6

[B92] Wu D. Raymond J. Wu M. Chatterji S. Ren Q. Graham J. E. (2009). Complete genome sequence of the aerobic CO-oxidizing thermophile *Thermomicrobium roseum*. *PLoS ONE* 4:e4207 10.1371/journal.pone.0004207 PMC2615216 19148287

[B93] Yabe S. Aiba Y. Sakai Y. Hazaka M. Yokota A. (2010). *Thermosporothrix hazakensis* gen. nov., sp. nov., isolated from compost, description of Thermosporotrichaceae fam. nov. within the class Ktedonobacteria Cavaletti etal. 2007 and emended description of the class Ktedonobacteria. *Int. J. Syst. Evol. Microbiol.* 60 1794–1801 10.1099/ijs.0.018069-0 19767365

[B94] Yabe S. Aiba Y. Sakai Y. Hazaka M. Yokota A. (2011). *Thermogemmatispora onikobensis* gen. nov., sp. nov. and *Thermogemmatispora foliorum* sp. nov., isolated from fallen leaves on geothermal soils, and description of Thermogemmatisporaceae fam. nov. and Thermogemmatisporales ord. nov. within the class Ktedonobacteria. *Int. J. Syst. Evol. Microbiol.* 61 903–910 10.1099/ijs.0.024877-0 20495028

[B95] Ying J.-Y. Zhang L.-M. He J.-Z. (2010). Putative ammonia-oxidizing bacteria and archaea in an acidic red soil with different land utilization patterns. *Environ. Microbiol. Rep.* 2 304–312 10.1111/j.1758-2229.2009.00130.x 23766082

